# Using chlorhexidine soap as an alternative lubricant for harvesting split skin grafts

**DOI:** 10.1308/003588412X13373405387096g

**Published:** 2012-05

**Authors:** JN Rodrigues, PM Geary

**Affiliations:** Nottingham University Hospitals NHS Trust,UK

Common lubricants for split skin graft harvesting include paraffin (which can cause persistent greasiness, preventing the use of adherent dressings to the donor site) or saline (which may not lubricate adequately, affecting graft quality). Alternatively, a weak dilution of chlorhexidine soap solution used for hand scrubbing, for example HiBiSCRUB® (Mölnlycke, Dunstable, UK), provides a more slippery surface than saline. After graft harvest, its water solubility allows it to be washed off easily prior to applying adhesive dressings to the donor site, unlike paraffin. By diluting it in saline (approximately 1 part in 200), foaming in the dermatome is avoided.

